# The Impact of SARS-CoV-2 Pandemic on Patients with Malignant Melanoma at a Romanian Academic Center: A Four-Year Retrospective Analysis

**DOI:** 10.3390/ijerph19148499

**Published:** 2022-07-12

**Authors:** Hazzaa Aabed, Vlad Bloanca, Zorin Crainiceanu, Felix Bratosin, Cosmin Citu, Mircea Mihai Diaconu, Ovidiu Ciorica, Tiberiu Bratu

**Affiliations:** 1 Department of Plastic Surgery, “Victor Babes” University of Medicine and Pharmacy Timisoara, 300041 Timisoara, Romania; haza_a_dr@hotmail.com (H.A.); crainiceanu.zorin@umft.ro (Z.C.); tiberiu.office@brol.ro (T.B.); 2 Methodological and Infectious Diseases Research Center, Department of Infectious Diseases, “Victor Babes” University of Medicine and Pharmacy, 300041 Timisoara, Romania; felix.bratosin7@gmail.com; 3 Department of Obstetrics and Gynecology, “Victor Babes” University of Medicine and Pharmacy Timisoara, Eftimie Murgu Square 2, 300041 Timisoara, Romania; citu.ioan@umft.ro (C.C.); diaconu.mircea@umft.ro (M.M.D.); 4 Business Administration and Economics Faculty, West University of Timisoara, Johann Heinrich Pestalozzi Street 16, 300115 Timisoara, Romania; ovidiu.ciorica@e-uvt.ro

**Keywords:** SARS-CoV-2, COVID-19, skin cancer, melanoma, epidemiology, plastic surgery

## Abstract

Considering cancer patients may be at an increased risk of severe COVID-19 disease, their oncologic treatment cannot be delayed without risking their oncologic outcomes. Considering this, a comprehensive evaluation is required for the management of malignant diseases such as melanoma. The current study aimed to assess the impact of the COVID-19 pandemic on the delivery of cancer care services for patients diagnosed with malignant melanoma in Romania; to document the difference in patients’ addressability and melanoma staging between the pandemic and pre-pandemic periods; as well as to determine the risk factors responsible for disease progression during the pandemic. We developed a retrospective analysis using a monocentric hospital database to compare the final 24 months of the pre-pandemic era to the first 24 months of the COVID-19 pandemic. All outpatients and inpatients with a diagnosis of malignant melanoma were screened during the study period and included in the analysis if matching the inclusion criteria. A total of 301 patients were included in the study, with 163 cases identified in the 24 months before the COVID-19 pandemic and 138 patients during the first 24 months of the pandemic. It was observed during the first two lockdown periods from March to May 2020, and, respectively, from October to December 2020, that significantly fewer patients with malignant melanoma presented for specialized medical care, while there was a statistically significantly lower proportion of outpatients due to COVID-19 restrictions (18.1% vs. 42.9%). The average Breslow depth was 1.1 mm before the pandemic, compared with 1.8 mm during the pandemic (*p*-value < 0.001). Third-stage patients were the most prevalent during both study periods, although with a statistically significant difference during the pandemic, with an increase from 90 (55.2%) patients to 94 (68.1%) (*p*-value < 0.001). The significant risk factors for disease progression were advanced AJCC stage (HR = 3.48), high Breslow index (HR = 3.19), postponed treatment (HR = 2.46), missed appointments (HR = 2.31), anemia at presentation (HR = 1.60), and patient’s age (HR = 1.57). After the pandemic limitations are brought to an end, a broad skin-cancer-screening campaign is warranted to detect the missed cases during COVID-19.

## 1. Introduction

On 26 February 2020, the first patient in Romania was identified with severe acute respiratory syndrome coronavirus 2 (SARS-CoV-2) infection [1]. The new highly pathogenic coronavirus is responsible for the acute respiratory disease known as coronavirus disease 19 (COVID-19), which was initially identified in late December 2019 in Wuhan, China [2]. In a short period, Romania and Europe were overrun by an unparalleled spread of the virus that transformed into a pandemic on 11 March 2020, according to the World Health Organization [3,4,5]. In two years, COVID-19 has killed almost two million patients in Europe, and over six million globally, as of May 2022 [6,7].

COVID-19 has drastically altered hospital treatment methods and workflows worldwide [8]. In Romania, the government imposed substantial limitations on social and public life to control the quick viral spread. Patients and medical workers were the main focus of the implementation of rigorous safety measures. Hospitals were organized to restrict elective treatments and prepare for the emergence of COVID-19 patients. As a result, in the majority of hospitals, equipment and personnel were shifted to COVID-19 patients’ care, and elective appointments or scheduled surgical procedures were postponed for many weeks [9]. These approaches have had a significant impact on dermatology and plastic surgery departments in several hospitals in Romania [10], where elective admissions were targeted for emergent interventions and oncological patients, while the personnel was transferred to assist in the care of COVID-19 patients. In general, this practice happened during the whole two-year pandemic, but mostly during the lockdown phases, which were marked by significant restrictions and a high number of COVID-19 hospital admissions. These preliminary results imply that the COVID-19 pandemic may have caused a significant drop in the frequency of skin cancer diagnoses during the pandemic waves and may have caused a delay in the treatment of skin cancer [11].

Particular populations, such as the elderly, pregnant women, and those with chronic diseases, are more susceptible to SARS-CoV-2 infection’s adverse effects [12,13,14,15]. According to current data, patients with malignancies are at a higher risk for life-threatening infections [16,17]. The presence of active cancer such as melanoma and chemotherapy for melanoma may reduce physical capacity and trigger immunosuppression, hence escalating the requirement for hospitalization and hospital visits and admissions [18]. All of these circumstances may increase the likelihood of COVID-19 infection and the development of severe consequences [19,20,21].

Melanoma represents the most lethal histology of skin cancer [22], where the vertical (Breslow) tumor thickness is a reliable prognostic factor. Increased Breslow thickness and tumor ulceration correspond with a poor prognosis because of the increased likelihood of metastasis [23]. Consequently, early diagnosis and surgical treatment of initial melanomas are crucial since timely diagnosis is critical for the survival of the patients [24]. Due to a probable decline in diagnoses of early-stage melanomas and delays in the presentation of patients with thicker tumors, the lower degree of access to medical treatment during COVID-19 poses a medical issue [25].

Centralized databases allow determining the impact of the COVID-19 pandemic on the epidemiology of melanoma and other skin cancers while also predicting outcomes based on missed diagnosis and appointments. An example from Italy, a country that was severely affected by SARS-CoV-2 during the early phases of the pandemic, where approximately 30 thousand cancer patients were analyzed, the highest excess mortality has been predicted in those with melanoma and female genital cancers [26]. In contrast, there is scant information on the number of missed cases and advanced cancer during the pandemic in less developed countries such as Romania, with the exception of a few monocentric studies or mathematical models that speculated on the possible effects of a lack of diagnostic facilities and missed medical checkups [27,28].

Therefore, the purpose of this research is to provide a series of data and real-world statistics involving patients with malignant melanoma from Romania during the COVID-19 pandemic. The main focus is to compare the pre-pandemic with the pandemic period, describing patients’ clinical features, cancer diagnosis and progression, and available treatment. The secondary end-point is to analyze the outcome of patients treated in our institute as risk factors for disease progression.

## 2. Materials and Methods

### 2.1. Study Design and Ethics

An observational study was conducted in the Department of Plastic Surgery of the Timis County Emergency Clinical Hospital “Pius Brinzeu” in Timisoara, Romania, which is a public institution associated with the “Victor Babes” University of Medicine and Pharmacy. The current study was designed based on a retrospective cohort design. The research population and relevant features were identified using a population-based administrative database and paper records of patients who were addressed to the outpatient and inpatient settings of the Department of Plastic Surgery throughout the study period. The centralized database contained patient medical records that were protected by privacy laws and obtained with patients’ agreement to include their medical history, investigations, and surgical and oncological data. The Ethics Committee of the “Victor Babes” University of Medicine and Pharmacy in Timisoara, Romania and the Ethics Committees of the Timis County Emergency Clinical Hospital “Pius Brinzeu” from Timisoara accepted the study’s design and protocol.

### 2.2. Inclusion Criteria and Study Variables

Adult patients older than 18 years who came for skin cancer treatment in an inpatient setting following a confirmed malignant melanoma diagnosis or in an outpatient setting for melanoma investigations and follow-up were included in the research between January 2018 and January 2022. The study aimed to compare the pre-pandemic period with the COVID-19 pandemic period. The pre-pandemic comprised the last 24 months from January 2018 until January 2020, while the pandemic period was considered as the time frame between January 2020 and January 2022. The first wave was considered as the period between March and October 2020, corresponding to a three-month lockdown in Romania, from March to May 2020 [29]. The second COVID-19 wave was between October 2020 and February 2021, when a two-month shutdown was imposed by the government between October and November 2020. The third pandemic wave in Romania was between February and July 2021, that came with lower levels of restrictions due to the onset of the COVID-19 vaccination campaign, while the fourth wave was between July 2021 and December 2021. Lastly, the fifth pandemic wave in Romania was between December 2021 and March 2022, that came without a lockdown or other important restrictions in the health sector [29].

As a tertiary clinic that is involved in treating malignant skin cancers, all patients were referred to the institution involved in the study through primary or secondary care referrals. All successive hospitalizations and those scheduled for investigations and regular follow-ups at the Plastic Surgery Department were included, provided they fit the inclusion criteria and study protocol. Patients whose tests and diagnoses were not validated and who lacked the requisite information or authorization to participate in the current study were excluded. A second criterion for exclusion was the lack of melanoma staging.

Melanoma staging is essential for recognizing the neoplastic stage, determining the extent of tumor cell invasion, and developing a definite patient profile. Using tumor–node–metastasis (TNM) melanoma staging, patients may be categorized into several stages, ranging from melanoma in situ (stage 0) to stage IV (metastatic melanoma). This staging system is important for determining the optimal treatment choices, prognosis, and survival rate. The American Joint Committee on Cancer (AJCC) staging of melanoma has emphasized the significance of the Breslow index (BI) in the medical treatment of melanoma as follows: Tis—the melanoma cells are found in the very top layer of the epidermis. T1—the melanoma is ≤1 mm in thickness. T2—the melanoma is between 1.1 mm and 2 mm-thick. T3—the melanoma is between 2.1 mm and 4 mm-thick. T4—the melanoma is >4 mm in thickness [30]. We used the eighth edition of the AJCC to assess the melanoma stages of study participants [31]. In addition to the presence or absence of ulceration, the mitotic rate of the tumor and the presence of tumor microsatellites in lymph nodes are other crucial TNM-staging factors.

Representatives of the clinical teams collected data on all malignant melanoma cases diagnosed during the study period that were anonymized prior to analysis. The following variables were gathered: (A). background data and baseline characteristics of the participants (age, age range, sex, body mass index, smoking history, place of origin, occupation, level of income, civil status, and history of SARS-CoV-2 infection); (B). malignant melanoma characteristics (inpatient/outpatient hospital service, comorbidities, melanoma clinical forms, Breslow index, anatomical distribution, AJCC TNM staging, and primary tumor ulceration); (C). outcomes and interventions (treatment methods used, referred for palliative care, reason for palliation, treatment complications, primary/secondary care referral source, referred to and received treatment, time from first signs until seeking medical opinion, change in the treatment plan, postponed treatment, missed appointments, ICU admission, and disease progression at three months).

### 2.3. Statistical Analysis

The software used for statistical analysis was MS EXCEL (Microsoft Corp. Redmond, Washington, DC, USA) and IBM SPSS version 27.0 (Armonk, NY, USA: IBM Corp.). A Kolmogorov–Smirnov test was used to determine the normality of the data. Continuous variables were expressed as the mean ± standard deviation (SD) or as the median with interquartile range (IQR). To compute the means and standard deviations, descriptive statistical analyses were conducted, while Student’s *t*-test was performed to determine the *p*-value. To analyze the differences in proportions, the chi-square and Fisher’s exact test were employed. A Cox regression model was built to determine factors that influence disease progression and included the variables with statistically significant differences between the two study periods. It was decided that a *p*-value of 0.05 was statistically significant.

## 3. Results

**Figure 1:**
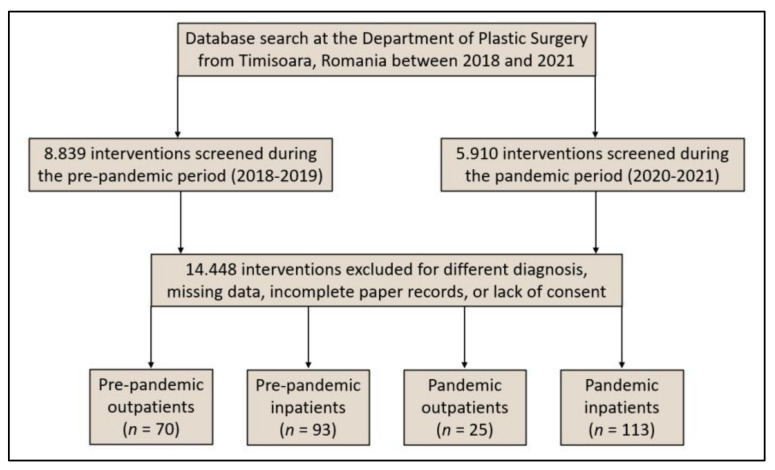
Flowchart displaying the inclusion process of patients with malignant melanoma during the 4-year study period.

Following the onset of the SARS-CoV-2 pandemic in Romania in March 2020 and the consequent implementation of lockdown precautions to prevent the spread of COVID-19, the number of patients with malignant melanoma diagnosis or suspicion addressing to specialized medical care has decreased significantly. Even if there was no reason to predict a sudden epidemiological change, this reduction was significantly different from the trend in the previous two years (2018 and 2019). Hence, the assumption supported by existing melanoma epidemiological data [32] is that, although it can develop quickly, without significant changes in the environmental factors such as UV exposure, there should not be any significant changes in its epidemiology [33]. Thus, the number of new malignant melanoma cases did not naturally decrease or remain equivalent to the year preceding the onset of the COVID-19 pandemic, but fewer of these new cases were effectively identified or observed in the outpatient setting during the pandemic timeframe, as seen in Figure 1.

**Figure 2:**
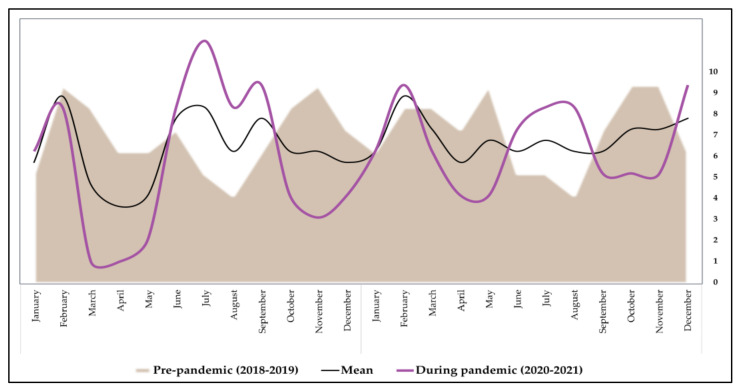
Evolution of malignant melanoma patient addressability before and during the COVID-19 pandemic. X-axis represents a monthly overlay of melanoma cases during the years 2018–2019 and 2020–2021. Y-axis represents the number of patients recorded each month.

Figure 2 provides a full profile of the patients who presented to our outpatient and inpatient clinic for malignant melanoma examination and treatment before and during the SARS-CoV-2 outbreak. It was observed during the first lockdown periods from March to May 2020 and, respectively, from October to December 2020 that significantly fewer patients with malignant melanoma presented for specialized medical care. Following the first lockdown, there was significant increase in the number of cases from June to September 2020, that is likely to be attributed to the patients that did not request medical care during the lockdown and decided to wait until an ease of restrictions. During the second year of the COVID-19 pandemic, the trends had a tendency to normalize.

### 3.1. Comparison of Baseline Characteristics

During the study period of 48 months, a total of 301 patients were selected by matching inclusion criteria, resulting in a group of 163 patients with malignant melanoma identified in the 24 months before the beginning of the COVID-19 pandemic, and another 138 patients identified during the first 24 months of the pandemic. Although there were slightly fewer cases that presented for specialized treatment in the plastic surgery department during the pandemic period, there was a statistically significantly lower proportion of outpatients due to COVID-19 restrictions when only emergencies were prioritized (18.1% pre-pandemic vs. 42.9% during the pandemic, *p*-value < 0.001) (Table 1).

The average patient was 58 years old (58.1 ± 16.3 years before the pandemic, respectively, and 58.8 ± 15.9 years during the pandemic), without statistically significant differences in proportions of gender, body mass index, chronic smoking history, and chronic alcohol use between the study groups. More than 45% of the patients in the cohort were in the 51 to 70 years old age range, and men were more commonly affected by the malignancy (53.4% before the pandemic, compared with 50.7% during the pandemic). The average body mass index in the full cohort was 26.1 kg/m^2^ (26.0 ± 3.8 before the pandemic, and 26.2 ± 4.1 during the pandemic), representing an overweight population. Although the plastic surgery department represented a large metropolitan and urban area, many patients residing in the rural regions were diagnosed (44.8% before the pandemic, compared with 37.0% during the pandemic). Since March 2020, a total of 26 (18.8%) have been infected with SARS-CoV-2.

### 3.2. Comparison of Clinical and Oncological Characteristics

Table 2 describes patients’ comorbidities and their cancer characteristics stratified by the period before and during the COVID-19 pandemic. It was observed that a majority of patients during both study periods were suffering from comorbid cardiovascular conditions, in an overall proportion of 43%, followed by respiratory disease in 24% of the entire cohort. However, there were no statistically significant differences between the two study groups regarding patient comorbidities. Melanoma of the trunk was the most typical anatomical distribution among the entire cohort, without significant differences (46.0% before the pandemic vs. 52.9% during the pandemic).

**Figure 3:**
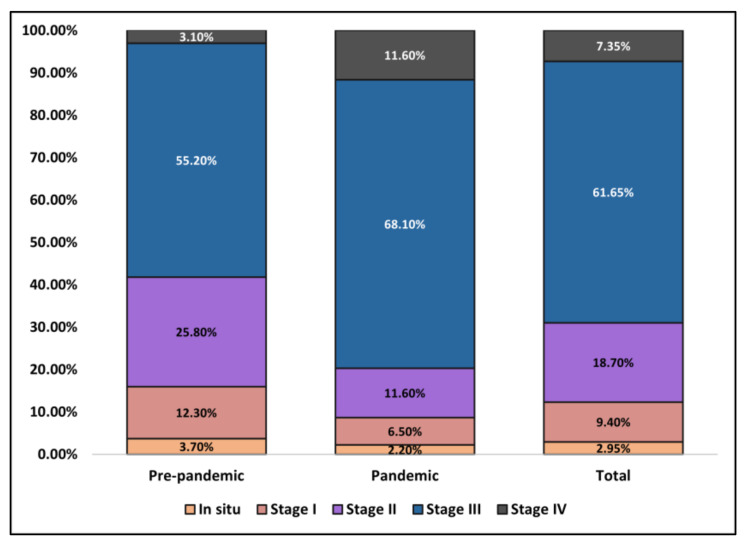
Comparison in AJCC malignant melanoma staging between patients seeking medical care before and during the COVID-19 pandemic.

The most common clinical form of melanoma was superficial spreading (66.3% before the pandemic and 67.4% during the pandemic), followed by nodular-type (28.2% before the pandemic and 27.5% during the pandemic), without any noteworthy differences. However, it was observed during the two years of the COVID-19 pandemic that the Breslow index of malignant melanoma cases was significantly different in proportions of depth. During 2018 and 2019, a total of 30.1% of patients were identified with a Breslow index between 1 and 2 mm, compared to 20.3% between 2020 and 2021, respectively, and five patients (3.1%) compared to 16 patients (11.6%) presented with a Breslow higher than 4 mm (*p*-value < 0.001). Additionally, the average Breslow depth was 1.1 mm before the pandemic, compared with 1.8 mm during the pandemic (*p*-value < 0.001). Similarly, patients were observed to present in later stages during the pandemic, as described by the AJCC TNM staging in Figure 3. Third-stage patients were the most prevalent during both study periods, although with a statistically significant difference during the pandemic, with an increase from 90 (55.2%) patients to 94 (68.1%) (*p*-value < 0.001). Lastly, tumor ulceration was present in 17.2% of patients before the pandemic, compared to 24.6% during the pandemic (*p*-value = 0.110).

### 3.3. Comparison of Outcomes and Interventions

Regarding the outcomes and interventions performed on the study population, it was observed that wide local excision was the most common procedure in approximately 90% of all patients, as seen in Table 3. However, during the pandemic, there were 12 (8.7%) patients with unresectable tumors, compared to a statistically significantly lower number of 4 (2.5%) patients before the pandemic (*p*-value = 0.038). Lymph node evaluation was performed by sentinel node biopsy or dissection of a lymph node group, with statistically significant differences between the two study periods (29.9% sentinel node biopsies before the pandemic vs. 16.0% during the pandemic, *p*-value = 0.038). Other statistically significant findings were the reasons for palliation referral, as the poor prognosis was determined in a higher proportion of patients during the pandemic (43.6% vs. 36.8%, *p*-value = 0.027). Moreover, the duration of hospitalization was also significantly higher during the pandemic (7.0 days vs. 5.9 days before the pandemic, *p*-value = 0.011).

Concerning treatment complications, anemia and depression were statistically more prevalent among the pandemic group (50.0% vs. 36.8%, *p*-value = 0.021), respectively (33.3% vs. 22.7%, *p*-value = 0.039). The referral source was for 103 (63.2%) patients before the pandemic from primary care, compared with 70 (50.7%) during the pandemic from secondary care (*p*-value = 0.025). It was also observed during the COVID-19 pandemic that a significantly higher proportion of patients waited longer until seeking their first medical opinion, from a median of 6 weeks to a median of 9 weeks (*p*-value < 0.001), while also postponing treatments more often (18.8% vs. 8.0%, *p*-value = 0.005), and missing more appointments (20.3% vs. 11.7%, *p*-value = 0.039). Lastly, disease progression at three months was statistically significantly higher during the COVID-19 pandemic, with 47 (34.1%) patients compared to 38 (23.3%) before the pandemic (*p*-value = 0.039).

### 3.4. Prognostic Factors

**Figure 4:**
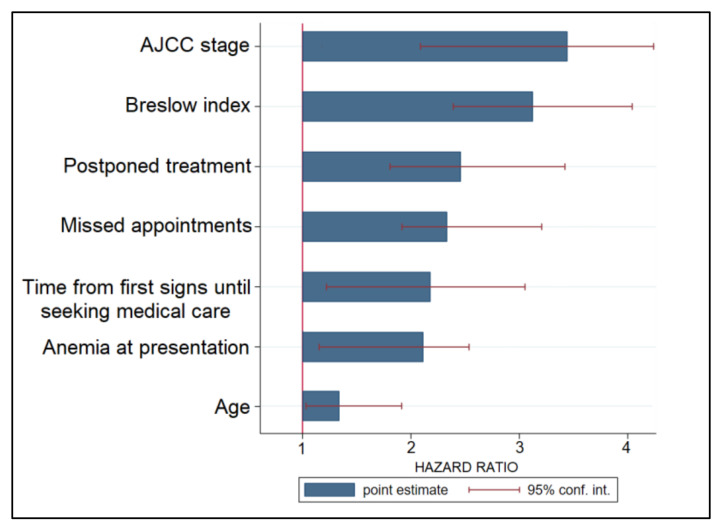
Risk factor analysis for disease progression in patients with malignant melanoma.

A Cox regression model to analyze risk factors for disease progression was performed and described in descending order of hazard ratios (HR) in Table 4 and Figure 4. The highest risk factor was an advanced AJCC stage, with patients having a 3.48 times higher likelihood of disease progression (*p*-value < 0.001), followed by a high Breslow index (HR = 3.19, *p*-value < 0.001). Other significant factors for disease progression were postponed treatments (HR = 2.46), missed appointments (HR = 2.31), the duration of waiting time from first signs until seeking medical opinion (HR = 2.18), and anemia at presentation (HR = 1.60); lastly, a nonmodifiable factor was the patient’s age (HR = 1.57, *p*-value = 0.030).

## 4. Discussion

### 4.1. Literature Findings

In the present investigation, it was established retrospectively how the COVID-19 pandemic in Romania affected the diagnosis and treatment of melanoma patients. These results match the majority of the modeling, prediction, and supposition that shows a significant number of cancer cases were overlooked throughout the continuing SARS-CoV-2 pandemic. In addition, a major concern is determined by the possibility that a significant number of patients who were initially diagnosed with skin cancer that was curable in the early stages became incurable in later stages due to skipped appointments, deliberately refused treatment, or intentionally delayed treatment due to fear of SARS-CoV-2 infection, as previously observed [34].

In this perspective, our findings are in accordance with other studies, such as an investigation performed in England during the first four months of the pandemic that indicated an almost 70 percent decline in skin cancer cases compared to the same time in the year before the pandemic [35]. Curiously, skin cancer waiting times decreased during COVID-19 relative to the same period before the pandemic, with a median of 8 days compared to 12 days, as reported before. These statistics demonstrate a statistically significant decrease in skin cancer diagnoses and wait times over the period covered by the COVID-19 pandemic. However, the results were not further described, such as by subtype (such as malignant melanoma (MM)), as presented in the current research. Another study of skin cancer in the United Kingdom revealed that almost half of the respondents had to delay Mohs micrographic surgery during these early months of the pandemic [36].

Similarly, an evaluation of pathology records from seven Italian pathology units found as well an almost 60% drop in all skin cancer diagnoses during the first months of the pandemic, compared to the pre-pandemic era, in addition to a 30% drop in surgical activity and a 55% decrease in new malignant melanoma diagnoses [37]. In addition, a multicenter study comparing the immediate post-lockdown period in the first 12 months of the pandemic to the average of the preceding 12 months revealed a 20 percent drop in MM diagnoses [38]. This finding suggests the high variability that occurred in different countries based on the locally imposed restrictions and healthcare performance. There were preliminary indicators of qualitative worsening with a higher detection rate of unfavorable tumors with greater Breslow tumor thickness, which was attributed to a delay in tumor identification. Similarly, a review of outpatients at a single institution in Germany revealed a considerable decrease in outpatient cases, particularly for malignant skin disorders during the first pandemic wave, as compared to prior years [39]. Additionally, certain patient categories, including those older than 85 years and those with malignant, chronic inflammatory, and infectious disorders, were determined to be more likely to be missing appointments during the pandemic.

Other risk factors for disease progression and negative outcomes in cancer patients, such as anemia, were analyzed in detail in different studies, as the existing literature describes [40]. In comparison to other patients, women and those with nodular melanoma histology, an increased erythrocyte sedimentation rate, a greater serum lactate dehydrogenase, lymph node involvement, and metastatic disease, were more likely to have low hemoglobin levels. Serum hemoglobin levels were not substantially correlated with age, anatomic localization, or numerous clinical characteristics, such as Breslow index, mitotic rate, or melanoma ulceration. The scientists discovered that hemoglobin levels were substantially related to cancer prognosis and that individuals with low hemoglobin concentrations had a worse survival rate than those with higher hemoglobin concentrations [41]. Although our study did not focus on patient survival, it was observed that anemia was an independent risk factor for disease progression.

The current study also did not focus on how SARS-CoV-2 infection and the COVID-19 vaccines might affect patients with melanoma. Switzer et al. [42] analyzed the primary problem as how the SARS-CoV-2 virus and vaccination could affect people with malignant melanoma receiving anti-PD-1 therapy as immune checkpoint inhibitors’ (ICI) anticancer and antiviral responses. The most recent guidelines state that the current pandemic is not a reason to discontinue this therapy, although ICI should be delayed during the active phase of the SARS-CoV-2 infection, and that patients on ICI should vaccinate against COVID-19. It was decided that patients should be made aware of the fact that many of our current clinical strategies are based on consensus rather than carefully monitored empirical evidence and that the current vaccination recommendations are based on individuals who have not received ICI treatment and do not have cancer.

### 4.2. Study Limitations and Future Perspectives

This research examines the epidemiology, public health, and clinical characteristics of patients with malignant melanoma from Romania. However, it is constrained by its retroactive design and the quality of the data digitally reproduced from patients’ paper records. A further limitation is the relatively small sample size imposed by our hospital database as a monocentric study; hence, the current findings may not entirely and accurately reflect the characteristics and outcomes of individuals with malignant melanoma in Romania during the COVID-19 pandemic. Lastly, it is important to mention the pandemic effects on the quality of hospital registries, as the risk of COVID-19 among healthcare workers was high; therefore, the risk of SARS-CoV-2 infection and the increased number of COVID-19 patients might have altered the registry capacity or the quality of data.

Nevertheless, the current study is solid evidence of the consequences and outcomes of COVID-19 for patients with malignant melanoma. The primary contribution is the consolidation of current information, and we advocate for the establishment of a threshold for an acceptable period of treatment deferral that does not impair future results and survival in patients suffering from melanoma. Therefore, multicentric and large sample studies are necessary to determine the entire range of effects triggered by the ongoing pandemic.

## 5. Conclusions

Although malignant melanoma is not one of the most frequent types of cancer, it is possible that many patients went undetected during COVID-19, while failing to do so will have long-lasting implications if these cases are not identified and addressed properly. Conclusively, some consultations may be postponed without serious implications, whilst others, particularly those concerning malignancies, must not be delayed in terms of precise diagnosis and quick treatment. After the pandemic limitations are brought to an end, a broad skin-cancer-screening campaign is warranted, as is one for the other prevalent malignancies identifiable by screening procedures.

## Figures and Tables

**Table 1:** Comparison of baseline characteristics of patients with malignant melanoma before and during the COVID-19 pandemic.

	Before COVID-19 (*n* = 163)	During COVID-19 (*n* = 138)	*p*-Value *
**Background**			
Age, years (mean ± SD)	58.1 ± 16.3	58.8 ± 15.9	0.707 **
**Age range**			0.874
≤30	9 (5.5%)	10 (7.2%)	
31–50	39 (23.9%)	36 (26.1%)	
51–70	76 (46.6%)	62 (44.9%)	
≥71	39 (23.9%)	30 (21.7%)	
**Sex**			0.646
Female	76 (46.6%)	68 (49.3%)	
Male	87 (53.4%)	70 (50.7%)	
BMI, kg/m^2^ (mean ± SD)	26.0 ± 3.8	26.2 ± 4.1	0.661 **
Chronic smoking history	37 (22.7%)	33 (23.9%)	0.803
Chronic alcohol use history	6 (3.7%)	5 (3.6%)	0.978
**Place of origin**			0.169
Rural	73 (44.8%)	51 (37.0%)	
Urban	90 (55.2%)	87 (63.0%)	
**Occupation**			0.335
Employed	102 (62.6%)	79 (57.2%)	
Unemployment	14 (8.6%)	19 (13.8%)	
Retired	47 (28.8%)	40 (29.0%)	
**Level of income**			0.452
Low	47 (28.8%)	49 (35.5%)	
Medium	95 (58.3%)	74 (53.6%)	
High	21 (12.9%)	15 (10.9%)	
**Civil status**			0.467
Married	128 (78.5%)	113 (81.9%)	
Single/Divorced/Widowed	35 (21.5%)	25 (18.1%)	
**Hospital service**			<0.001
Outpatient	70 (42.9%)	25 (18.1%)	
Inpatient	93 (57.1%)	113 (81.9%)	
SARS-CoV-2 infection	-	26 (18.8%)	-

* Chi-square or Fisher’s exact test; ** Student’s *t*-test; SD, standard deviation.

**Table 2:** Malignant melanoma characteristics of patients before and during the COVID-19 pandemic.

	Before COVID-19 (*n* = 163)	During COVID-19 (*n* = 138)	*p*-Value *
**Comorbidities**			
Cardiovascular	71 (43.6%)	59 (42.8%)	0.888
Metabolic	26 (16.0%)	21 (15.2%)	0.861
Autoimmune	8 (4.9%)	6 (4.3%)	0.818
Respiratory	38 (23.3%)	34 (24.6%)	0.788
Renal	14 (8.6%)	16 (11.6%)	0.385
Digestive	13 (8.0%)	9 (6.5%)	0.629
Other	5 (3.1%)	7 (5.1%)	0.375
**Melanoma clinical forms**			0.958
Superficial spreading	108 (66.3%)	93 (67.4%)	
Nodular	46 (28.2%)	38 (27.5%)	
Lentigo maligna	4 (2.5%)	4 (2.9%)	
Acral lentiginous	5 (3.1%)	3 (2.2%)	
**Breslow index**			0.001
In situ	6 (3.7%)	3 (2.2%)	
<1 mm	31 (19.0%)	13 (9.4%)	
1–2 mm	49 (30.1%)	28 (20.3%)	
2.1–4 mm	72 (44.2%)	78 (56.5%)	
>4 mm	5 (3.1%)	16 (11.6%)	
Breslow index average, mm	1.1 ± 0.4	1.8 ± 0.5	<0.001
**Anatomical distribution**			0.528
Trunk	75 (46.0%)	73 (52.9%)	
Limbs	59 (36.2%)	45 (32.6%)	
Head and neck	20 (12.3%)	16 (11.6%)	
Extremities	90 (5.5%)	4 (2.9%)	
**AJCC TNM staging**			<0.001
0 (In situ)	6 (3.7%)	3 (2.2%)	
I	20 (12.3%)	9 (6.5%)	
II	42 (25.8%)	16 (11.6%)	
III	90 (55.2%)	94 (68.1%)	
IV	5 (3.1%)	16 (11.6%)	
**Primary tumor ulceration**			
Absent	135 (82.8%)	104 (75.4%)	0.110
Present	28 (17.2%)	34 (24.6%)	

* Chi-square or Fisher’s exact test; AJCC, American Joint Committee on Cancer.

**Table 3:** Outcomes and interventions of patients with malignant melanoma before and during the COVID-19 pandemic.

	Before COVID-19 (*n* = 163)	During COVID-19 (*n* = 138)	*p*-Value *
**Surgical treatment**			0.038
Mohs micrographic surgery	5 (3.1%)	3 (1.4%)	
Wide local excision	154 (94.5%)	124 (89.9%)	
Unresectable	4 (2.5%)	12 (8.7%)	
**Lymph node evaluation**			0.038
Sentinel node	23 (29.9%)	13 (16.0%)	
Dissection	54 (70.1%)	68 (84.0%)	
**Lymph node dissection region**			0.297
Axilla	38 (49.4%)	44 (54.3%)	
Inguinal	26 (33.8%)	30 (37.0%)	
Other zones	13 (16.9%)	7 (8.6%)	
**Skin repair**			0.342
Direct suture	119 (73.0%)	102 (73.9%)	
Skin graft	8 (4.9%)	9 (6.5%)	
Skin flap	24 (14.7%)	23 (16.7%)	
Free tissue transfer	12 (7.4%)	4 (2.9%)	
**Referred for palliative care**	38 (23.3%)	39 (28.3%)	0.326
**Reason for palliation**			
Poor prognosis	14 (36.8%)	17 (43.6%)	0.546
Distant metastasis	5 (13.2%)	12 (30.8%)	0.062
Poor performance status	19 (50.0%)	10 (25.6%)	0.027
Days of hospitalization	5.9 ± 3.8	7.0 ± 3.7	0.011 **
**Treatment complications**			
Local infection	22 (13.5%)	26 (18.8%)	0.207
Skin necrosis	5 (3.1%)	6 (4.3%)	0.555
Lymphoedema	19 (11.7%)	23 (16.7%)	0.211
Digestive	48 (29.4%)	52 (37.7%)	0.130
Anemia	60 (36.8%)	69 (50.0%)	0.021
Leucopenia	14 (8.6%)	15 (10.9%)	0.504
Depression	37 (22.7%)	46 (33.3%)	0.039
**Referral source**			0.025
Primary care	103 (63.2%)	68 (49.3%)	
Secondary care	60 (36.8%)	70 (50.7%)	
**Outcomes**			
Time from first signs until seeking medical opinion, weeks, median (IQR)	6 (5)	9 (7)	<0.001
Change in treatment plan	25 (15.3%)	34 (24.6%)	0.042
Postponed treatment	13 (8.0%)	26 (18.8%)	0.005
Missed appointments	19 (11.7%)	28 (20.3%)	0.039
ICU admission	3 (1.8%)	6 (4.3%)	0.203
Disease progression at 3 months	38 (23.3%)	47 (34.1%)	0.039

* Chi-square or Fisher’s exact test; ** Student’s *t*-test; IQR, interquartile range (percentile 25–percentile 75); ICU, intensive care unit.

**Table 4:** Risk factors for melanoma progression after the initial hospital visit.

Risk Factors	HR	CI	*p*-Value
AJCC stage	3.48	2.13–4.30	<0.001
Breslow index	3.19	2.36–4.08	<0.001
Postponed treatment	2.46	1.72–3.41	<0.001
Missed appointments	2.31	1.80–3.26	<0.001
Time from first signs until seeking medical opinion	2.18	1.13–3.15	0.001
Anemia at presentation	1.60	1.09–2.49	0.018
Age	1.57	1.04–1.94	0.030

AJCC, American Joint Committee on Cancer; HR, hazard ratio; CI, confidence interval.

## Data Availability

The data presented in this study are available on request from the corresponding author.
